# Hemoperfusion techniques using Jafron HA330 cartridge combined with BBraun Dialog+ dialysis machine in patient with coronavirus disease 2019 pneumonia and septic shock: a case report

**DOI:** 10.1186/s13256-023-03851-y

**Published:** 2023-04-08

**Authors:** Adrian Hartomuljono, Adhrie Sugiarto, Jennefer

**Affiliations:** Department of Anesthesiology and Intensive Care, Faculty of Medicine, Universitas Indonesia, Cipto Mangunkusumo National General Hospital, Jakarta, Indonesia

**Keywords:** Hemodialysis, Hemoperfusion, Septic shock, COVID-19, Chronic kidney disease

## Abstract

**Background:**

The use of hemoperfusion for cytokine removal and inflammatory mediators is increasingly intense, especially in coronavirus disease 2019 patients who are already known to the general public for having cytokine storms. However, we have known about these cytokine storms for a long time in the critical care world. One of the modalities to remove cytokines is to use filtration and adsorption techniques with continuous renal replacement therapy. The use of continuous renal replacement therapy is usually constrained by its very high cost compared with standard care, especially in Indonesia, where health costs are covered by national health insurance. In this case, we use hemodialysis and hemoperfusion, using a dialysis machine, which is more cost-effective and easy to use.

**Case presentation:**

We used the Jafron HA330 cartridge, modified for the BBraun Dialog+ dialysis machine. This case report presents an 84-year-old Asian man with septic shock due to pneumonia, congestive heart failure, and acute chronic kidney disease accompanied by fluid overload. After undergoing hemodialysis and hemoperfusion separately, there was a gradual and significant clinical improvement. Clinical indicators, including the vasopressor inotropic score and infection markers, should all be considered when deciding whether to begin hemodialysis and hemoperfusion.

**Conclusion:**

In general, using hemoperfusion to treat septic shock patients can reduce the length of stay in the intensive care unit, and morbidity and mortality.

## Background

Coronavirus disease 2019 (COVID-19) is a global disease outbreak, and about 5% of COVID-19 patients require appropriate therapy, intensive care, and complex disease management [1]. Sepsis is the most common cause of patient hospitalization in the intensive care unit. Although the prevalence of sepsis is increasing, somehow sepsis mortality has decreased. This is because the early detection and treatment of sepsis has progressed rapidly [2]. The Surviving Sepsis Campaign recommends fluid resuscitation, the use of vasopressors, and immediate administration of antibiotics, especially in the first 3 hours, as the main management of sepsis [3]. Extracorporeal blood purification has been suggested to enhance outcomes in sepsis patients by removing inflammatory mediators or bacterial toxins from the blood, which can favorably modify the host’s inflammatory response. There are several techniques to purify the blood itself, one of which is hemoadsorption. During hemoadsorption, sorbents directly contact with blood via an extracorporeal circuit through hydrophobic interactions, ionic attraction, hydrogen bonding, and van der Waals interactions—the sorbent draws solutes [4]. Jafron HA330 for hemoperfusion is a cost-effective, beneficial, and efficient approach for treating septic patients, particularly in settings with limited resources [5].

## Case presentation

An 84-year-old Asian male was referred to the emergency department (ED) due to his need for an intensive care room with hemodialysis. The patient was previously treated at another hospital with acute respiratory distress syndrome (ARDS) due to COVID-19, septic shock, electrolyte imbalance, uremic encephalopathy, acute chronic kidney disease (ACKD), and diabetes mellitus. The patient was previously treated with a high-flow nasal cannula (HFNC), remdesivir 100 mg once daily for 5 days, meropenem 1 g once daily, moxifloxacin 400 mg once daily, dexamethasone 5 mg once daily, as well as hemodynamic support drugs norepinephrine 0.15 µg/kgBW/minute and dobutamine 3 µg/kgBW/minute. The patient with motor aphasia due to stroke complications since 2016.

At ED, the patient was apathetic, Glasgow Coma Scale (GCS) was 13–14, blood pressure was 111/56 mmHg with norepinephrine and dobutamine, pulse ranged from 90 to 100 beats per minute, afebrile, and 97% oxygen saturation with nasal cannula 2 L per minute. On physical examination, crackles were found, especially on bilateral lung bases, peripheral edema was observed in all extremities, accompanied by involuntary movements of the extremities, and fluid overload was detected, with a central venous pressure (CVP) of 11 mmHg and thoracic fluid content (TFC) of 40 (normal value 25–35). The patient’s diuresis was < 0.3 cc/kgBW/hour. On further investigation, atrial fibrillation had normal ventricular response, lactate was 3.0, procalcitonin (PCT) was 162 ng/mL, C-reactive protein (CRP) was 60.8 mg/dL, and N-terminal pro-brain natriuretic peptide (NT-proBNP) was > 9000, with urea at 281 mg/dL and creatinine at 5.69 mg/dL. Electrolyte examination showed the following: sodium 150 mmol/L, potassium 4.9 mmol/L, and chloride 111 mmol/L. The cardiac echocardiography examination revealed an ejection fraction of 49%, and the computed tomography (CT) scan of the thorax presented bilateral pneumonia.

From the physical examination and supporting examination, our patient presented with septic shock due to pneumonia, congestive heart failure with fluid overload signs, electrolyte imbalance, and uremic encephalopathy due to ACKD. We planned fluid removal with continuous furosemide, and hemodialysis with the sustained low-efficiency dialysis (SLED) technique for 6 hours. We did not do hemoperfusion in conjunction with hemodialysis due to hemodynamic concerns. The patient was given piperacillin–tazobactam 4.5 g twice daily and vancomycin 1 g once daily. We continued using norepinephrine and dobutamine for hemodynamic support, with mean arterial pressure (MAP) target of 60–65 mmHg.

**Fig. 1:**
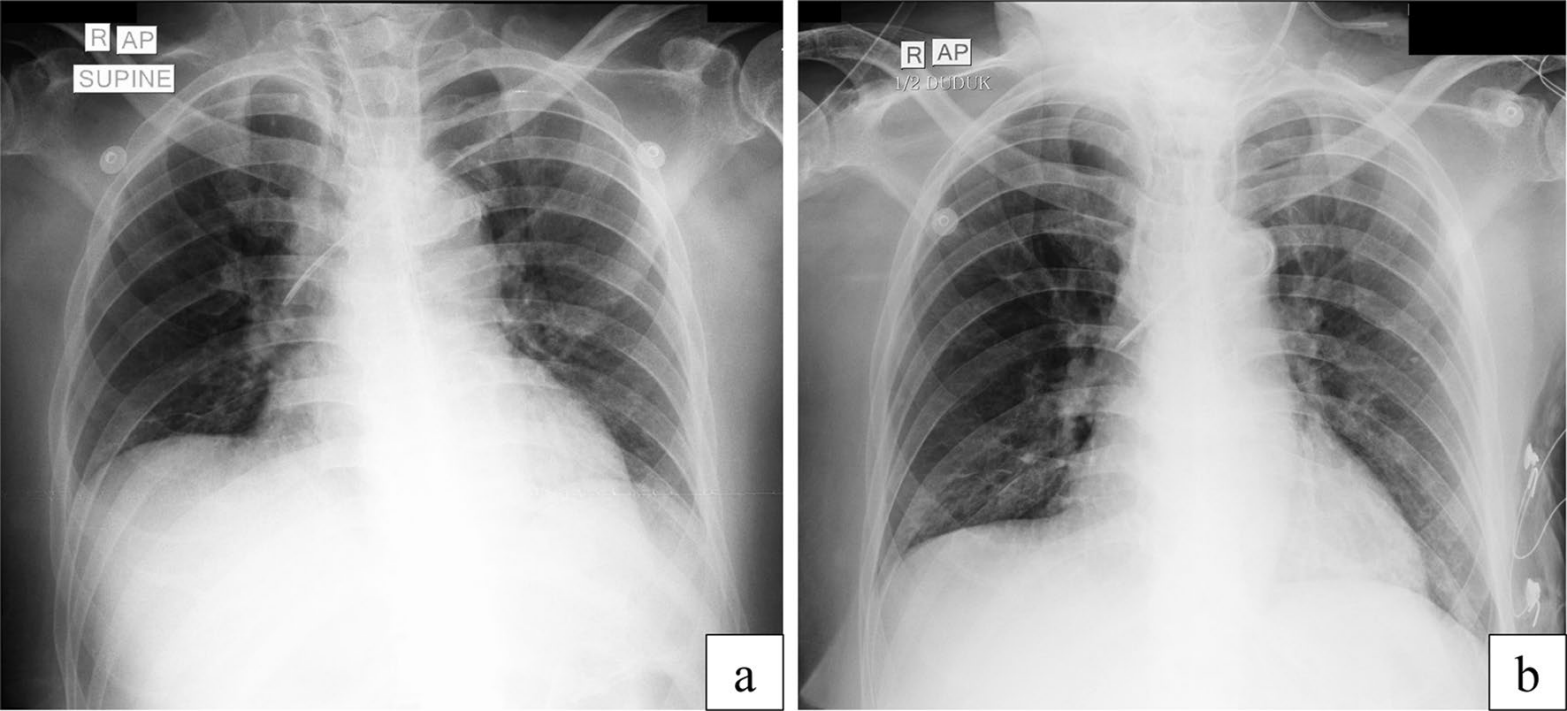
Patient’s chest x-ray when admitted to the emergency department (**a**), and after hemodialysis and hemoperfusion (**b**)

During hemodialysis, blood pressure fluctuates with quick blood (qB) of 100–150 mL/hour and ultrafiltration (UF) of 1000 mL/6 hours. After hemodialysis, the clinical condition began to improve, starting with an improvement of consciousness and diuresis. Also, on chest x-ray analysis, the infiltrates in both lung fields were in remission, compared with analysis before hemodialysis (Fig. 1). Urine output increased to 0.5–0.8 cc/kgBW using furosemide 2 mg/hour. However, there was an increasing need to increase norepinephrine to 0.5 µg/kgBW/minute, dobutamine to 7.5 µg/kgBW/minute, and vasopressin to 0.03 units/minute. Our expected end point here is a CVP target of 0–4 mmHg; clinically, no signs of peripheral edema were found.

**Fig. 2:**
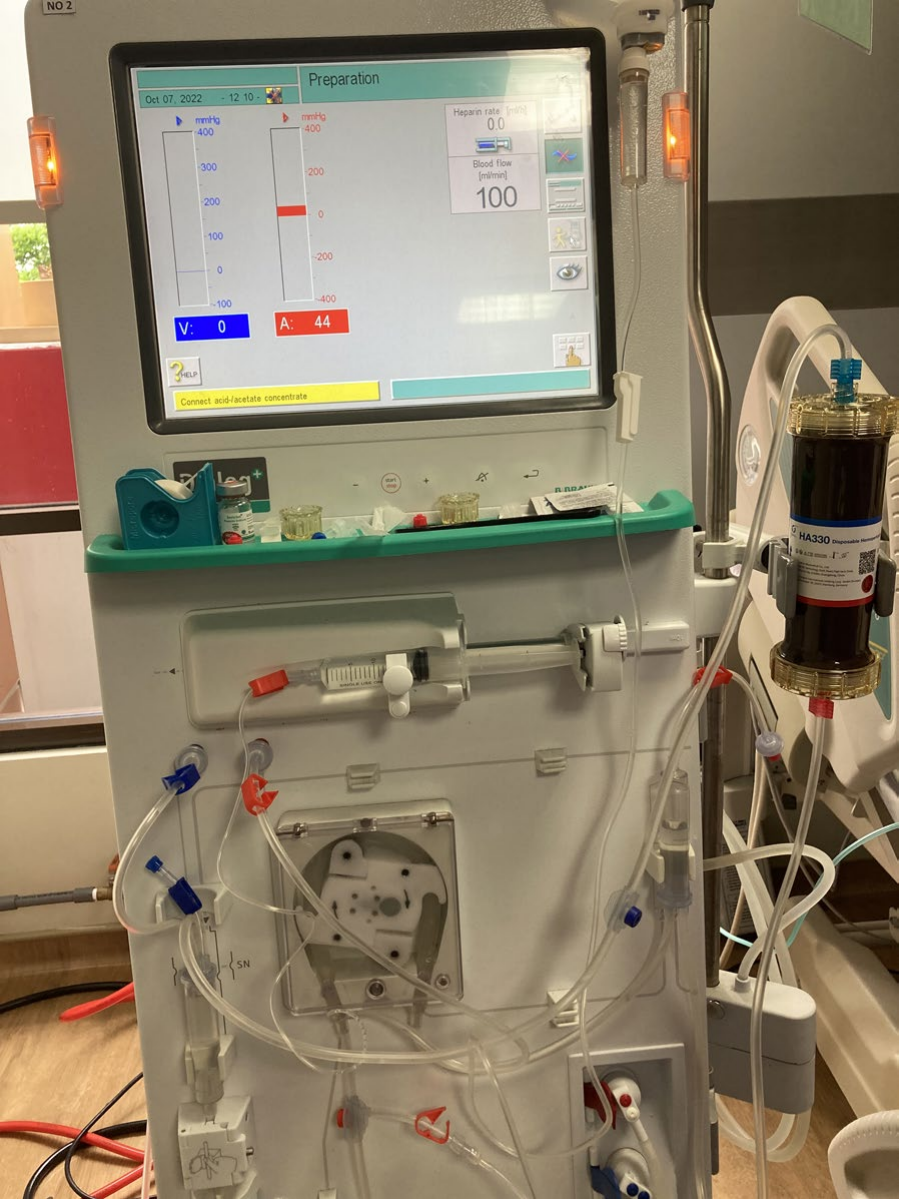
Jafron HA330 attached to BBraun Dialog+

At this point, after escalation, antibiotic therapy failed to decrease vasopressor requirements. Then treatment was continued with hemoperfusion using a Jafron HA330 cartridge, with a modified technique, in the BBraun Dialog+ dialysis machine (Fig. 2) with blood flow of 150 mL/hour, zero dialysate flow rates, and intermittent heparin bolus at the rate of 500 units every hour. The progress of the patient’s clinical improvement was seen 1 hour after we started hemoperfusion, which was characterized by an improvement in consciousness up to GCS 15, an increase in diuresis up to 1 cc/kgBW/hour, a decrease in vasopressor use with norepinephrine up to 0.2 µg/kgBW, dobutamine up to 2 µg/kgBW and vasopressin up to 0.02 units/hour, and an improvement in patient’s laboratory results (Table 1). We did not proceed to the next hemoperfusion session due to significant improvements.

**Table 1:** Laboratory parameters before and after hemodialysis–hemoperfusion

	Before HD and HP	After HD and HP	Normal value
Procalcitonin (ng/mL)	162.92	7.68	< 0.5
NT-proBNP (pg/mL)	> 9000	1458	< 400
CRP (mg/dL)	60.8	18	< 5
Urea (mg/dL)	281	132	10–50
Creatinine (mg/dL)	5.69	2.8	< 1.4
Sodium (mEq/L)	150	142	135–145
Potassium (mEq/L)	4.9	3.4	3.5–4.5
Chloride (mEq/L)	111	106	90–110
Hemoglobin (g/dL)	9.2	7.9	12–16
Leukocyte (per mm^3^)	7080	11,890	4000–10,000
Platelet (per mm^3^)	179,000	110,000	150–400,000
d-dimer (g/L)	2.2		< 0.5
Lactate (mmol/L)	3	1.2	< 2
NLR	16.6	8.9	4.0
CVP (mmHg)	11	3.5	

*NT-proBNP* N-terminal pro-brain natriuretic peptide, *CRP* C-reactive protein, *NLR* neutrophil–lymphocyte ratio, *CVP* central venous pressure

**Fig. 3:**
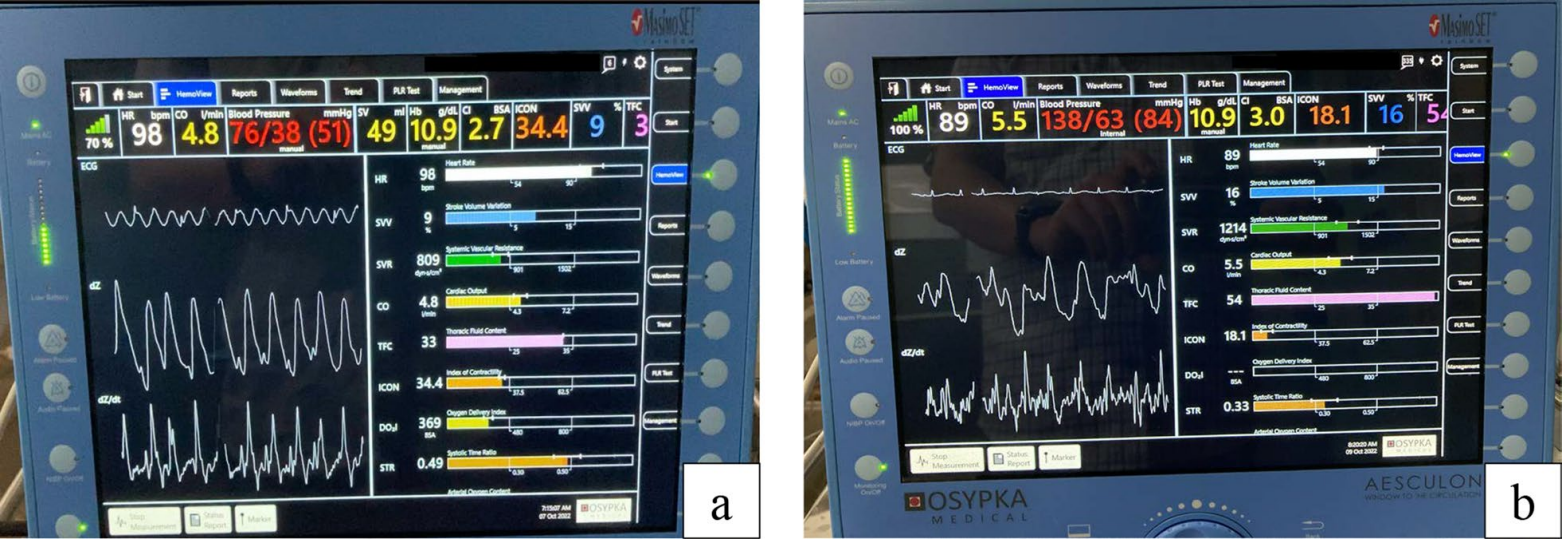
Hemodynamic evaluation before running hemoperfusion (**a**), and 2 hours after running hemoperfusion, using Aesculon (**b**)

We performed non-invasive monitoring of the patient’s hemodynamics (Fig. 3) before, and 2 hours after, initiating hemofiltration.

We adjusted the dosage of piperacillin–tazobactam to 4.5 g every 6 hours and vancomycin to 1 g every 12 hours after a decrease in creatinine level (2.8 mg/dL). Provision was made for 230 mL packed red blood cell transfusion, with a target hemoglobin (Hb) > 9.0 g/dL. Furosemide 2 mg/hour was still given to maintain a negative target balance with a CVP of 0–4 mmHg.

**Fig. 4:**
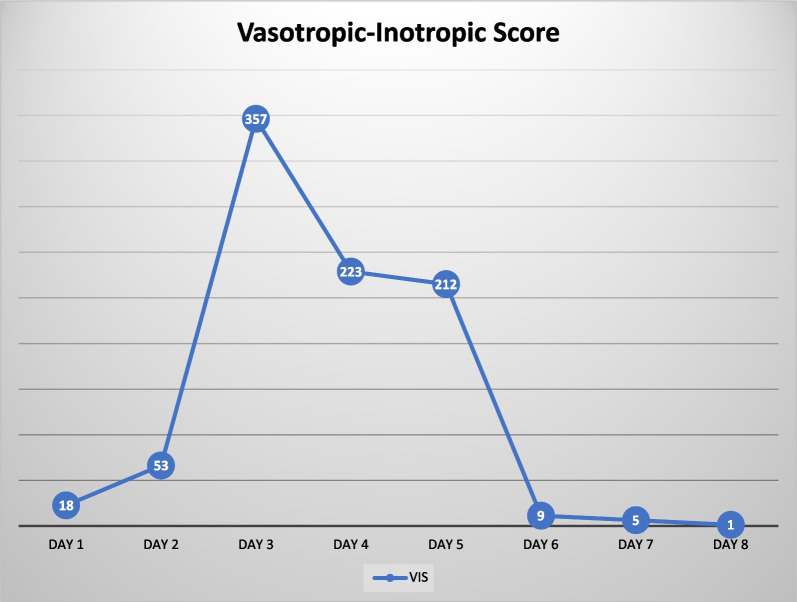
Vasotropic inotropic score (VIS) of the patient from day 1—intensive care unit admission, day 2—hemodialysis, day 3—hemoperfusion, day 4—antibiotic with total dose adjustment until day 8 when the patient was moved to the ward

The use of vasopressors was gradually decreased until it stopped on day 8 of treatment in the intensive care unit (ICU) (Fig. 4); the cumulative balance while in the ICU was −5.6 L and the CVP was 2 mmHg. The patient moved to the intermediate care room and continued the medical rehabilitation. The patient’s condition was still weak; however, he managed to respond to his family’s voice and the doctor’s questions.

## Discussion

Sepsis is a clinical and complex biological syndrome, defined as life-threatening organ dysfunction caused by an irregular host response to infection [6]. The cytokine storm is a potentially fatal systemic inflammatory state caused by high amounts of circulating cytokines and immune cell hyperactivation from sepsis [7]. The patient was treated aggressively with adequate antibiotics at first, yet remained hemodynamically unstable with worsening septic parameters. According to pneumonia care guidelines from the American Thoracic Society (ATS), it was decided to switch antibiotic therapy to piperacillin–tazobactam combined with vancomycin. We adjusted the dose based on the creatinine level and the patient’s history of involuntary movements.

The patient presented with fluid overload based on clinical condition and CVP measurements, and a rise in NT-proBNP value. We initiated fluid evacuation with furosemide and dialysis to enhance tissue perfusion. At this point, increased fluid administration is hazardous to the patient. Contrary to what some assume, peripheral and anasarca edema harms the patient because both can cause organ dysfunction [8]. Guyton’s idea of hemodynamic physiology demonstrates that venous return depends on the value of CVP; the lower the value of CVP, the more significant the difference between CVP and mean circulatory filling pressure (MCFP), and consequently, the same applies to venous return and cardiac output. To achieve an ideal venous return to the right heart, the CVP must be lower than the MCFP. High CVP will increase back pressure and impede venous return so that the systemic venous system will be injured (congestive organs) [9]. We aimed for a CVP between 0 and 4 mmHg in these patients.

After the hemodialysis session, urine production increased, indicating a clinical improvement. However, the demand of vasopressors rose, so we decided to adjust the antibiotic dosage and perform hemoperfusion. Extracorporeal blood purification, also known as hemoperfusion, is one of the therapy techniques that can be utilized to decrease cytokine levels. Hemoperfusion is an extracorporeal procedure involving blood passing through a cartridge containing sorbent material that removes solutes through direct binding. Hemoperfusion operates via an adsorption mechanism related to the different cartridges included in its construction. It differs from hemodialysis in that hemodialysis works by a diffusion process [10].

## Conclusion

Hemoperfusion using Jafron HA330 is easy, safe, and cost-effective. In addition to the Jafron hemoperfusion device, we utilized the BBraun Dialog+ dialysis machine. So, in the case of sepsis in this patient, other than de-resuscitation, blood purification techniques using hemoadsorption could enhance the patient’s overall state.

## Data Availability

The data that support the findings of this study are not publicly available. Data, however, are available from the authors upon reasonable request and with permission.

## Abbreviations

ACKD: Acute chronic kidney disease
ARDS: Acute respiratory distress syndrome
ATS: American Thoracic Society
BW: Body weight
CHF: Congestive heart failure
COVID: Coronavirus disease
CRP: C-reactive protein
CRRT: Continuous renal replacement therapy
CT: Computed tomography
CVP: Central venous pressure
ED: Emergency department
GCS: Glasgow Coma Scale
HD: Hemodialysis
HFNC: High flow nasal cannula
HP: Hemoperfusion
ICU: Intensive care unit
MAP: Mean arterial pressure
MCFP: Mean circulatory filling pressure
NLR: Neutrophil lymphocyte ratio
PCT: Procalcitonin
qB: Quick blood
SLED: Sustained low-efficiency dialysis
TFC: Thoracic fluid content
UF: Ultrafiltration
